# Alternating capture of attention by multiple visual working memory representations

**DOI:** 10.1038/s41598-023-40095-8

**Published:** 2023-08-10

**Authors:** Lunbo Zhang, Yuki Yamada

**Affiliations:** 1 Graduate School of Human-Environment Studies, Kyushu University, 744 Motooka, Nishi-ku, Fukuoka, 819-0395 Japan; 2 Faculty of Arts and Science, Kyushu University, Fukuoka, Japan

**Keywords:** Human behaviour, Psychology, Object vision

## Abstract

**Abstract:**

Can we look for multiple objects simultaneously? Previous studies have proposed that the representation of an item in visual working memory (VWM) can bias the deployment of attention to memory-matching items in visual search. However, it is still controversial whether multiple VWM item representations are able to capture attention. In the present study, we adopted an eye-tracking technique to reveal this issue. In Experiment 1, we replicated Chen and Du’s Experiment 2 and adopted an eye-tracking technique to determine whether multiple VWM item representations are able to bias attention. In Chen and Du’s paradigm, the memory test was always followed by the search test, and the participants might intentionally prepare for the search task, which can affect the results of the research. Thus, in Experiment 2, we prevented participants from predicting the temporal occurrence of the visual search task by randomly conducting a search test or a memory test after memoranda. The findings of the present study suggested that only one working memory item at a time influences attention and multiple working memory items may affect attention through alternation.

**Protocol registration:**

The stage 1 protocol for this Registered Report was accepted in principle on September 5, 2022. The protocol, as accepted by the journal, can be found at: 10.17605/OSF.IO/BE529.

## Introduction

In our daily life, we often look for multiple objects at the same time, such as finding certain files on a messy desktop, and finding the goods we need in a store. Although the experience of life suggests that we can look for multiple objects at the same time, the question remains—is it truly simultaneous? For example, if we want to search for files A and B on the desktop simultaneously, we can constantly switch targets between A and B, as if we are focusing on them at the same time.

Before answering this question, we need to know how to search for a target. Many studies have proposed that visual working memory (VWM) can bias the deployment of attention to memory-matching items in visual search^1,2^. People can maintain 3–4 working memory items, which may mean that we can search for 3–4 targets at the same time^3^.

However, a previous study showed that only one visual working memory representation can capture attention^4,5^. Furthermore, Olivers’ research suggests that there are two types of VWM representations^6^. One type is an active memory item that has direct access to perception, and thus can catch attention. The other type refers to accessory memory items, which are passively stored in VWM, and exert little influence on attention during visual search. Olivers’ research also proposed that only one item in the VWM at a time can serve as an active memory item. Another study showed that when search targets vary from trial to trial, it is assumed that only one search target can serve as an active memory item and consume the only slot in VWM during a visual search^7^. Thus, other accessory working memory items which are presented as distractors cannot interfere with concurrent visual search because only the active item can capture attention.

However, when the search target is constant from trial to trial, this representation is eventually transferred from VWM to long-term memory. This allows another item in VWM to become an active memory item and guide attention^8,9^. There are also studies showing that it is possible to search for two different targets at the same time^10,11^. However, limitations to the possible targets could cause VWM representations to be transferred to long-term memory more quickly^8,9,12^. Therefore, it is possible that these findings do not demonstrate two active representations of VWM.

The above studies have shown that even if presented as an interference stimulus, only one working memory item can become an active memory item and guide attention^8,9^. Is it true that only one active item can affect attention?

However, Chen and Du’s study presented two memory items which served as distractors in the search test. They found that each of the memory items can affect the reaction, and the interference is even greater than in one memory item condition. They proposed that although it cannot be used as a target stimulus, multiple memory items can be used as interference stimuli to guide attention^13^. King’s research successfully replicated these findings and further showed that three representations can directly influence attention^14^.

Why is there such a dispute? Most previous studies used reaction time (RT) as an experimental indicator. RT can be influenced by cognitive control, which can reverse the effect of memory-driven attentional capture^15,16^. In contrast, eye movement indicators, such as the first fixation proportion (FFP), are not easily affected by cognitive control; they are a more reliable dependent variable of the attentional guidance effect in the early stages of visual search^17^. Furthermore, Ort proposed that visual search has three stages: 1. preparation (establishing and maintaining a mental representation of a search target), 2. selection (using this mental representation to extract candidate targets from visual input), and 3. post-selection processing (verifying that the selected information is actually a target)^18^. Ort also proposed that in the race for selection, template-matching objects have a competitive advantage and drive the eyes to themselves. In other words, eye movement indicators reflect the effect of preparation and interaction of top-down biases with visual input leading up to selection. RT reflects a series of processes including verifying whether the selected object is indeed the target after the selection stage. Eye movements are therefore more responsive to the process of “capturing attention” than RT.

However, existing studies that measured eye movements show inconsistent results^17,19,20^. In Zhang’s study, participants were asked to memorise two items after which they were shown the items in an in-sequence recognition experiment^17^. The authors assumed that the stimulus that came first would remain in active status, while the stimulus that came later would remain in accessory status. Their study found that the stimuli that came later could also capture attention, therefore, they concluded that multiple VWM representations—even the accessory representation—can simultaneously interact with visual attention. Nevertheless, as participants are asked to recognise both memory items, we cannot confirm that the stimulus that comes later is entirely stored as an accessory representation; Zhang’s study is based on Olivers’ assumption that one item must be an active representation and other items will be accessory representations, however, this assumption does not necessarily apply to their visual search task. That is, the logic of assuming that one item will become an accessory representation and that studying this item will lead to the conclusion that multiple items can guide attention at the same time has certain limitations. Beck’s research used another method to explore this question and found that there was no significant difference in oculomotor capture between the multiple-item condition and the one-item condition^20^. When participants remember multiple items, only one of the multiple items can guide attention; therefore, there is no significant difference in the effect on attention between multiple-item and one-item conditions. However, Beck’s study used only a limited set of 4 colors as stimuli; the limited possible targets could cause the two VWM representations to be quickly transferred to long-term memory^8,9,12^. Therefore, these findings may not demonstrate two representations of VWM.

In Experiment 1, we used Chen’s paradigm to prevent working memory from transferring to long-term memory^13^. This paradigm used twelve possibilities composed of two features (colour and texture) that vary from trial to trial; therefore, memory items should be maintained as VWM representations^13^. Based on this, the FFP was set to detect the early stages of visual search. To test whether the VWM in one-item condition can capture attention, we compared the VWM item condition with the non-VWM item (the item which is not presented before) condition. The same analysis was applied to either VWM in the two-item condition. If the VWM can capture attention, the FFPs on the VWM item would be significantly higher than the FFPs on non-VWM item. We estimated whether VWM representations can be simultaneously activated to capture attention by calculating the interference caused by distractors and comparing the interference in the one-item condition with the interference in the two-item condition. If there is only one active VWM slot, the two VWM representations can be alternately activated to guide attention across trials, and the stimulus would have a 1/2 chance of being an active item; therefore, a 1/2 chance of capturing attention. This meant that the combined memory-driven capture effect of two-item condition should be equal to the effect of one-item condition. If there are multiple active VWM slots, then both VWM representations can be activated simultaneously to guide attention, and irrelevant distractors matching either of the two VWM representations would also capture attention. Therefore, for two VWM representations in two-item condition, the combined memory-driven capture effect should be significantly larger than that of one-item condition (illustrated in Table 1). In Chen and Du’s paradigm, the memory items were always followed by the search test, which meant that the participants may intentionally prepare for the search task. As the subsequent visual search test was independent of memory items, working memory items are suppressed in advance, resulting in memory items not being well templated in the preparation phase of visual search. Eye movements reflect the effect of preparation and interactions of top-down biases with the visual input leading up to selection^18^; this may lead to an underestimation of the impact of memory items on visual search tests. In Experiment 2, each trial randomly conducted one of two tests (search test or memory test) after the memoranda were shown in an unpredictable manner, preventing participants from predicting the temporal occurrence of the visual search task (illustrated in Table 1). Participants cannot predict the temporal occurrence of the visual search task, and participants cannot prevent templating in advance, thereby preventing underestimation of the impact of working memory.

**Table 1:** Design table.

Question	Hypothesis (if applicable)	Sampling plan (e.g. power analysis)	Analysis plan	Interpretation given to different outcomes
When RT and FFP serve as dependent variables, do simultaneous multiple visual working memory representations indeed capture attention?	1. Multiple visual working memory representations simultaneously capture attention	We will recruit 24 participants through Kyushu University	A memory-capture index (MCI) was calculated to measure the interference caused by distractors. We will run a paired-samples *t*-test on the number of memory items (one vs. two)	1. The sum interference of two distractors in two memory items condition is significantly larger than the interference in one memory condition
	2. Only one working memory representation can capture attention			2. The sum interference of two distractors in two memory items condition is not significantly different from the interference in one memory condition
Do participants intentionally prepare for the search task, thus affecting the results of the research?	1. Yes	We will also recruit 24 participants through Kyushu University	We will run the same analysis as Experiment 1	1. In Experiment 1, we find that multiple visual working memory representations simultaneously capture attention, however, the results of Experiment 2 show that only one working memory representation can capture attention
	2. No			2. Both experiments show that multiple visual working memory representations simultaneously capture attention

## Methods

### Ethics information

The experiment was conducted in accordance with the guidelines of the Declaration of Helsinki^21^. The present study received approval from the psychological research ethics committee of the Faculty of Human-Environment Studies at Kyushu University, Fukuoka, Kyushu, Japan (approval numbers: 2021-017 and 2022-010), and informed consent was obtained from all participants. Participants received 1000 yen as a reward.

### Design

#### Experiment 1

##### Materials and equipment

The experiment was controlled using the MATLAB R2022a with Psychtoolbox 3.0.17 on a Precision 3630 Tower Dell desktop computer with a 27-in. ASUS VG278QR-R Gaming Monitor (100 Hz refresh rate) at a viewing distance of approximately 60 cm. Eye movements were recorded using Tobii Pro Nano.

##### Visual task description and procedure

At the beginning of each block the participants performed a standard five-point calibration. An adjustable chin rest helped to maintain head position.

Memoranda. In each trial, two circles were presented with a 2.5° visual angle to the left and right of fixation with a radius of 0.6° for 500 ms that the participants were asked to remember. 12 possible colour-texture combinations of four colours (red (RGB: 250, 20, 0; luminance: 70.6 cd/m^2^; Chromaticity Coordinates: 0.642, 0.341), green (RGB: 0, 170, 0; luminance: 87.1 cd/m^2^; Chromaticity Coordinates: 0.291, 0.638), yellow (RGB: 220, 200, 20; luminance: 205 cd/m^2^; Chromaticity Coordinates: 0446, 0.510), or blue (RGB: 0, 90, 200; luminance: 17.8 cd/m^2^; Chromaticity Coordinates: 0.158, 0.110)) and three types of texture (checkerboard: each square on the checkerboard was a square with a side length of 3°, striped: Each stripe is 0.17° wide and had a spatial frequency of 2.92 c/deg, and reticulation: each circle was 0.09° in diameter, and the distance between the centres of each circle is 0.18°) were randomly chosen as the circles.

In the one-item condition, participants were asked only to memorize the Cued item which was pointed by a grey arrow (RGB: 85, 85, 85; luminance: 3.80 cd/m^2^; Chromaticity Coordinates: 0.345, 0.356; 0.8° in width, 1.6° in length) above two circles. In contrast, no arrow appeared in the two-item condition, indicating that participants should memorise both circles.

Search. After an interval of 1000 ms (a fixation presented in the centre of the screen that participants were asked to stare at), there was a search display. The search display consisted of a grey diamond (1.2° in size) and seven circle distractors (each with a radius of 0.6°). They were placed on the rim of an imaginary circle (with a radius of 8°), which was centred on the fixation. Six of the seven disks were grey circles, and the other one was chosen from 12 possible combinations of colour and texture. The diamond contained a black target letter which could be either an “N” or an “M” (0.38° in size). Participants were instructed to indicate whether the diamond contained an “N” or an “M” as fast as possible, by hitting the “N” or an “M” key on the keyboard. The search display remained on the screen until participants selected “N” or an “M” Each circle distractor contained a symbol resembling an hourglass.

Memory test. After a 300 ms blank screen, eight probe disks were displayed, and participants were instructed to reply whether the memorised disks were present in the eight probe disks. If the memorised disks were present in the eight probe disks, participants should hit the “N” key or if the memorised disks were not present in the eight probe disks participants should hit the “M” key. Probe circles could share the same colour but differ in texture or shared the same texture but differed in colour as the memorised item; therefore, participants cannot use a single feature for a memory task. In the one-item condition, the Cued item only appeared in half of the trials, and the Uncued item was never displayed as a probe circle. However, in the two-item condition, the M1 and M2 items were present in the probes with equal probability for 50% of the trials. They did not occur simultaneously in the probe display.

**Figure 1:**
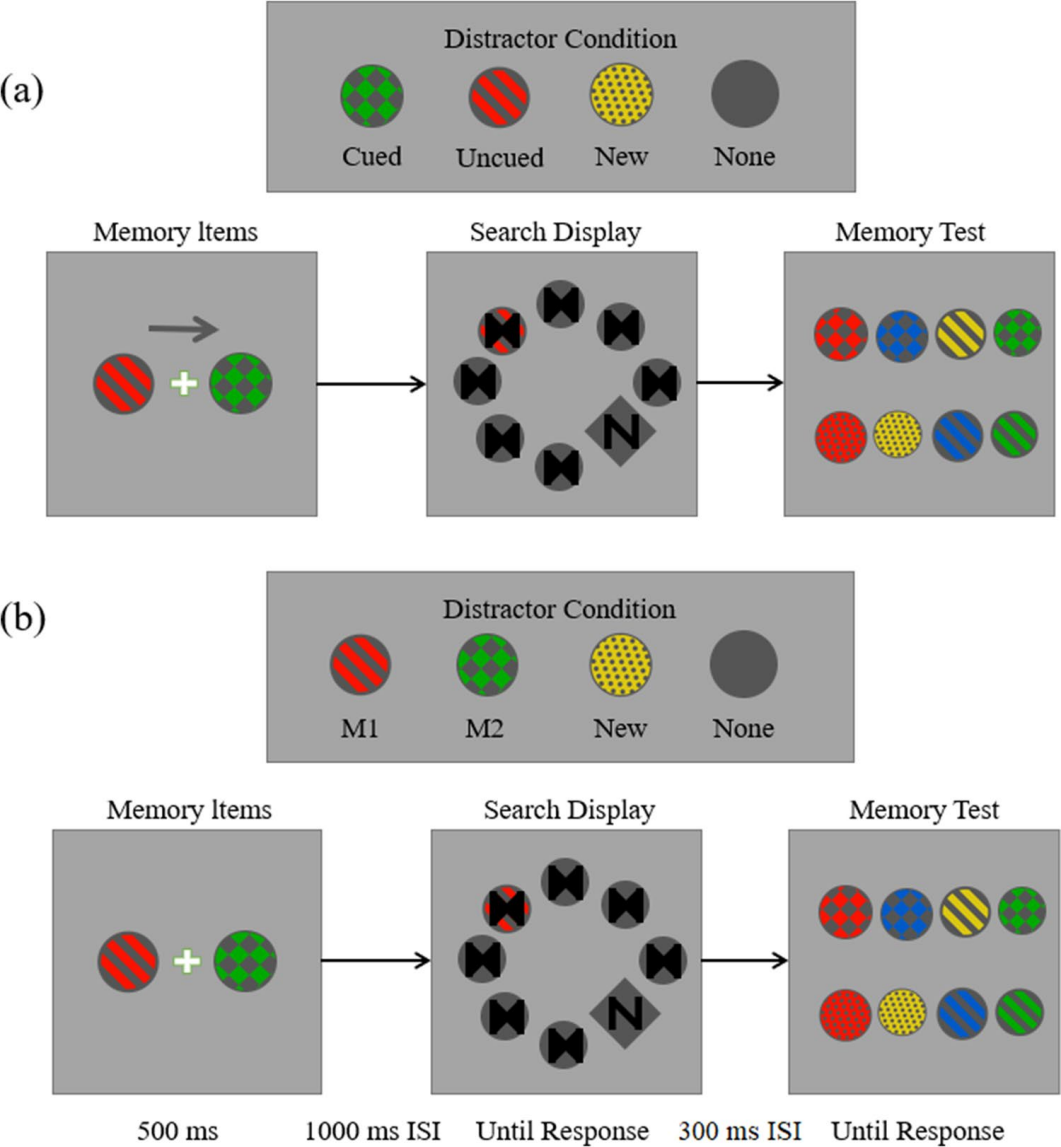
Trial sequences for Experiment 1. (**a**) Each trial had four different possible distractor conditions for the one-item memory condition (the “Cued distractor”: the circle’s colour and texture were the same as Cued memorized item; the “Uncued distractor”: the circle’s colour and texture were the same as the uncued memorized item; the “New distractor”: the distractor circle was a different colour and texture from the 12 possible combinations of colour and texture; the “None distractor”: no distractor circle appeared, all seven circles were grey). The number of trials for each condition was the same and the sequence order was random. The singleton distractor appeared randomly in eight positions and all locations were equally likely to contain the distractor. (**b**) Each trial had four different possible distractor conditions per trial for the two-item memory condition (the “M1 distractor”: the circle’s colour and texture were the same as the memorized item on the left; the “M2 distractor”: the circle’s colour and texture were the same as the memorized item on the right; the “New distractor” and “None distractor” conditions were the same as their counterparts in the one-item memory condition). The number of trials for each condition was the same and the sequence order was random. The singleton distractor appeared randomly in eight positions and all locations were equally likely to contain the distractor.

Task procedure. The order of the two memory conditions was counterbalanced across participants. Each participant completed two one-item memory sets and two two-item memory sets. In the one-item condition, the four distractor conditions were Cued, Uncued, New, and None. In the two-item condition, the four distractor conditions were: M1, M2, New, and None (illustrated in Fig. 1). These conditions were counterbalanced. There were 24 practice trials and two blocks of 96 trials for each of the two memory conditions (48 trials per distractor condition). In practice trials, we provided feedback at the end of each trial on whether the participant’s eyes focused their eyes on the center of the screen at the beginning of the search display.

Eye-tracking data was analysed with respect to one region of interest: the critical distractor region (a circle with radius 1.6°; this represents a deviation from our Stage 1 research plan which specified a 1.2° radius, with reasons for this expansion detailed in the “Discussion” section).

#### Experiment 2

##### Materials and equipment

The experiment was controlled using the MATLAB R2022a with Psychtoolbox 3.0.17 on a Precision 3630 Tower Dell desktop computer with a 27-in. ASUS VG278QR-R Gaming Monitor (100 Hz refresh rate) at a viewing distance of approximately 60 cm. Eye movements were recorded using Tobii Pro Nano.

##### Visual task description and procedure

**Figure 2:**
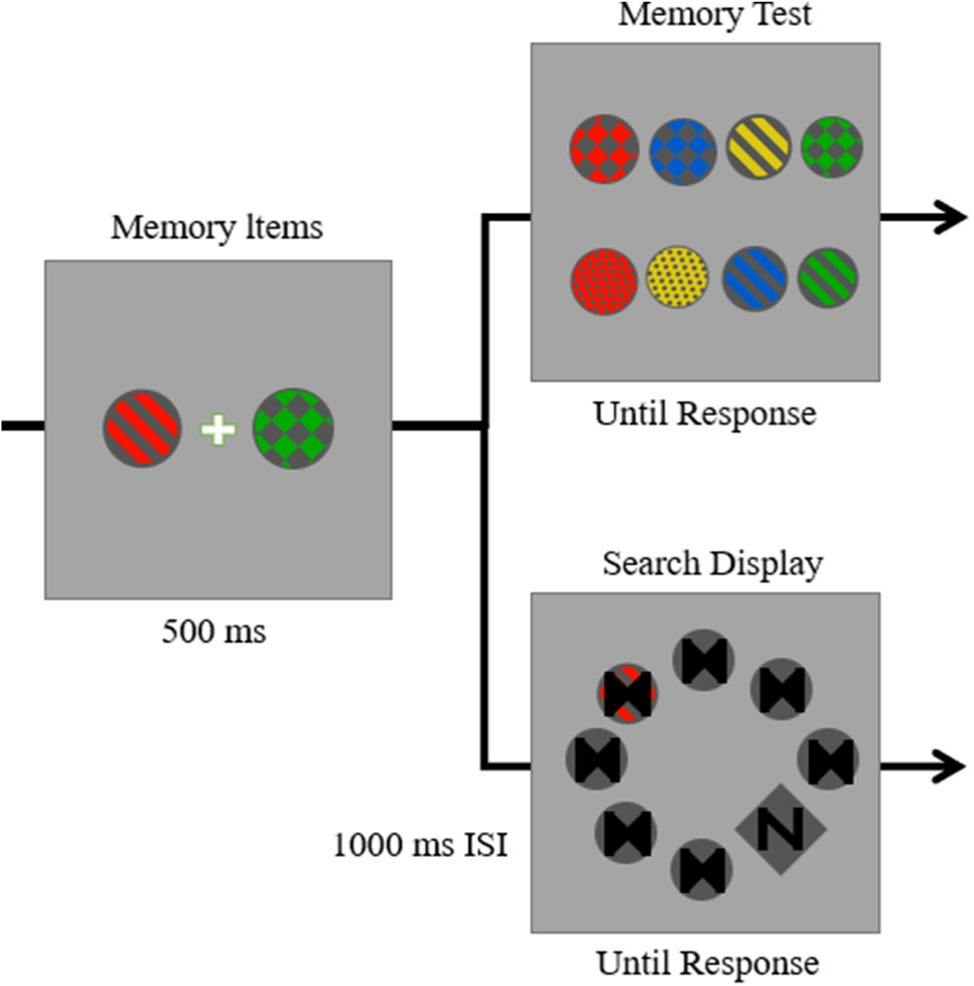
Trial sequences for Experiment 2. All experimental conditions were the same as those of Experiment 1.

Except for the following points, these methods were the same as those in Experiment 1. In half of the trials, the memoranda test was followed by the memory test; in the other half, the memoranda test was followed by the search test (illustrated in Fig. 2). As doing so would halve our data, we doubled the trials to ensure that we obtained the same amount of data. There were 24 practice trials and four blocks of 192 trials for each memory condition (because half of the trials only reply whether the memorised disks were present, without search display. Therefore, there were still kept 48 trials per distractor condition).

### Sampling plan

We recruited 24 participants separately in each experiment at Kyushu University. We excluded ineligible participants according to the exclusion criteria (described later) until 24 participants had been recruited. Before the start of the experiment, we ensured that the participants could clearly distinguish each stimulus. Participants' data were removed when their accuracy on the memory task is below 60%. Participants must provide written informed consent before participating in the study.

### Analysis plan

In each experiment, participants’ data were rejected when their accuracy in the memory task was below 60%. When computing the FFP, trials were eliminated from further analysis based on the following criteria: (1) Trials in which the first gaze was not located in the region of central fixation (4° around the central fixation; this represents a deviation from our Stage 1 research plan which specified a 3° radius, with reasons for this expansion detailed in the “Discussion” section). (2) The target object was not fixed during the search. (3) Eye-movement data were unavailable. (4) If the search time on a trial (elapsed time until target fixation) was more than ± 2.5 SD from the participant’s condition mean. Participants in whom more than 40% of their trials met the exclusion criteria were excluded from the analysis. We used the MATLAB EyeMMV toolbox to analyse eye movement data^22^. The spatial parameter was t1 = 0.250, and the minimum fixation duration was 150 ms.

We calculated the mean of each person's RTs and the FFPs, and analysed these two dependent variables. FFP here meant the proportion of trials, in which the first fixation fell within the ROI relative to the total number of trials in the respective condition. We performed a one-way repeated-measures ANOVA separately for one-item condition (Cued, Uncued, New, and None) and two-item condition (M1, M2, New, and None). Further multiple comparisons were performed. By comparing the different memory item condition (Cued, M1 or M2) with the New item condition, we confirmed whether the memory item can guide attention. If VWM can capture attention, the FFPs on distractor in the memory item condition will be significantly higher than that in the New item condition. As exploratory analyses, we also examined any trends across conditions from the other multiple comparison results.

A memory-capture index (MCI) was calculated to measure the interference caused by distractors^13^. It was calculated as follows:

$$MCI\; Cued = \frac{FFP\; Cued - FFP \;New}{{0.5\left( {FFP \;Cued + FFP \;New} \right)}}$$

$$MCI \;M1 \left( {or M2} \right) = \frac{{FFP\; M1 \left( {or M2} \right) - FFP\; New}}{{0.5\left( {FFP\; M1 \left( {or M2} \right) + FFP\; New} \right)}}$$

The MCI for the two-item condition was equal to the sum of MCI M1 and MCI M2. The MCI for the one-item condition was equal to MCI Cued. We ran a paired-samples *t*-test on the number of memory items (one vs. two).

## Results

### Experiment 1

According to the registered exclusion criteria, four participants were excluded because their memory test accuracy was no better than 60%. A total of 12 participants were excluded from the analysis because 40% or more trials were invalid under the established exclusion criteria. The analyses were based on data from the remaining participants (11 men and 13 women, mean age = 21.83 years).

#### Reaction time

Table 2 presents the descriptive statistics. ANOVA with a Greenhouse–Geisser correction showed that the main effect of the distractor condition in the one-item memory condition was significant, *F*(3, 69) = 5.45, *p* = 0.008. Multiple comparisons with the Holm-Sidak method revealed that the RTs in the Cued distractor condition were significantly larger than the RTs in the None distractor condition, *t*(23) = 5.16, *p* < 0.001, Cohen’s *d* = 0.20.

**Table 2:** RTs (ms) for Experiment 1’s visual search task across all conditions.

Memory condition	Distractor condition	Mean (SD)
Single-item memory	Cued	1731 (631)
	Uncued	1629 (565)
	New	1628 (571)
	None	1532(566)
Two-item memory	M1	1957 (737)
	M2	1934 (738)
	New	1883 (707)
	None	1786 (603)

In addition, the main effect of the distractor condition on the two-item memory condition was significant, *F*(3, 69) = 5.21, *p* = 0.003. Multiple comparisons with the Holm-Sidak method revealed that the RTs in the M1 and M2 distractor conditions were significantly larger than the RTs in the None distractor condition, *t*(23) = 4.36, *p* = 0.001, Cohen’s *d* = 0.17, and *t*(23) = 2.96, *p* = 0.034, Cohen’s *d* = 0.15.

#### FFPs

**Figure 3:**
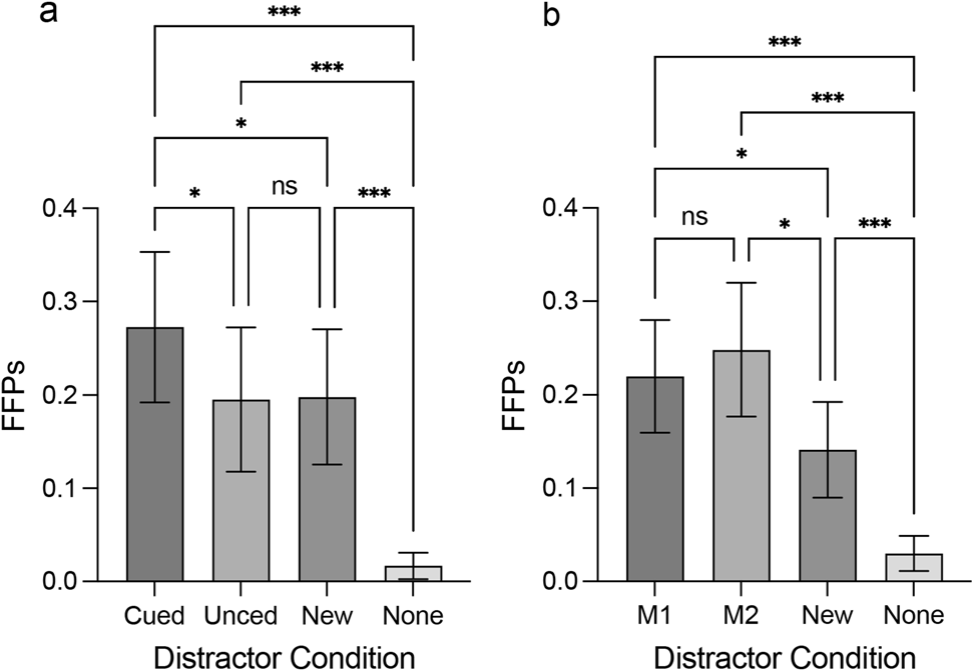
Results of Experiment 1. (**a**) FFPs for the search display as a function of the distractor condition in the one-memory item condition. (**b**) FFPs for the search display as a function of the distractor condition in the two-item memory condition. Error bars indicate 95% CI. *indicates *p* < 0.05, ** < 0.01, *** < 0.001.

Tables 3 and 4 present the descriptive statistics. ANOVA with a Greenhouse–Geisser correction showed a main effect of the distractor condition in the one-item memory condition, *F*(3, 69) = 23.30, *p* < 0.001. Multiple comparisons with the Holm-Sidak method revealed that the FFPs from the Cued distractor condition were significantly larger than the FFPs of the Uncued distractor condition (*t*(23) = 3.20, *p* = 0.012, Cohen’s *d* = 0.08), the New distractor condition (*t*(23) = 2.42, *p* = 0.047, Cohen’s *d* = 0.07), and the None distractor condition (*t*(23) = 6.72, *p* < 0.001, Cohen’s *d* = 0.26). The FFPs from both the Uncued distractor and New distractor conditions were significantly larger than the FFPs from the None distractor condition, *t*(23) = 4.74, *p* < 0.001, Cohen’s *d* = 0.18, and *t*(23) = 5.14, *p* < 0.001, Cohen’s *d* = 0.18, respectively (see Fig. 3a).

**Table 3:** FFPs for Experiment 1’s visual search task across all conditions.

Memory condition	Distractor condition	Mean (SD)
Single-item memory	Cued	0.27 (0.19)
	Uncued	0.20 (0.18)
	New	0.20 (0.17)
	None	0.02 (0.03)
Two-item memory	M1	0.22 (0.14)
	M2	0.25 (0.17)
	New	0.14 (0.12)
	None	0.03 (0.04)

**Table 4:** MCI for Experiment 1’s visual search task across all conditions.

Memory condition	Distractor condition	Mean (SD)
Single-item memory	MCI cued	0.45 (0.83)
Two-item memory	MCI M1	0.43 (0.92)
	MCI M2	0.51 (0.95)

There was also a main effect of the distractor condition in the two-item memory condition, *F*(3, 69) = 23.14, *p* < 0.001. Multiple comparisons with the Holm-Sidak method revealed that the FFPs from the M1 distractor condition were significantly larger than the FFPs from the New distractor condition and the None distractor condition, *t*(23) = 3.07, *p* = 0.012, Cohen’s *d* = 0.08, *t*(23) = 6.57, *p* < 0.001, Cohen’s *d* = 0.19. The M2 distractor condition was significantly larger than the FFPs from the New distractor condition and the None distractor condition, *t*(23) = 3.20, *p* = 0.012, Cohen’s *d* = 0.11, and *t*(23) = 6.55, *p* < 0.001, Cohen’s *d* = 0.22. The New distractor condition was significantly larger than the FFPs from the None distractor condition, *t*(23) = 4.69, *p* < 0.001, Cohen’s *d* = 0.11 (see Fig. 3b).

Memory capture index (MCI) was calculated to measure the interference caused by distractors. Experiment 1’s MCI analysis supported the following: the difference between the combined MCI effect of the M1 and M2 distractors in the two-item memory condition and that of the Cued distractor in the one-item memory condition was not significant, *t*(23) = − 1.24, *p* = 0.226, Cohen’s *d* = − 0.49. These results suggest that participants alternated between M1 and M2 as the sole active representation.

Based on the high exclusion rate this time, this might have an impact on our results. As an unregistered exploratory analysis, we included all data regardless of the exclusion criteria. The results of the FFPs were almost identical to those of the data filtered using the exclusion criteria. Only the difference between M1 and New condition was nonsignificant. See the Dataset for further details.

### Experiment 2

One participant was excluded because a memory test accuracy was no better than 60%. A total of 9 participants were excluded from the analysis because 40% or more trials were invalid under the established exclusion criteria. The analyses were based on data from the remaining participants (15 men and 9 women, mean age = 25.58 years).

#### Reaction time

Table 5 presents the descriptive statistics. ANOVA with a Greenhouse–Geisser correction showed that the main effect of the distractor condition in the one-item memory condition was significant, *F*(3, 69) = 17.45, *p* < 0.001. Multiple comparisons with the Holm-Sidak method revealed that the RTs in the Cued distractor condition were significantly longer than the RTs of the New distractor condition and the None distractor condition, *t*(23) = 4.18, *p* = 0.001, Cohen’s *d* = 0.15, and *t*(23) = 5.96, *p* < 0.001, Cohen’s *d* = 0.21. The RTs in the Uncued distractor condition were significantly longer than the RTs of the New distractor condition and the None distractor condition, *t*(23) = 2.64, *p* = 0.043, Cohen’s *d* = 0.09, and *t*(23) = 8.77, *p* < 0.001, Cohen’s *d* = 0.16.

**Table 5:** RTs (ms) for Experiment 2’s visual search task across all conditions.

Memory condition	Distractor condition	Mean (SD)
Single-item memory	Cued	1474 (382)
	Uncued	1422 (411)
	New	1328 (308)
	None	1264 (389)
Two-item memory	M1	1405 (342)
	M2	1444 (432)
	New	1440 (531)
	None	1230 (293)

In addition, the main effect of the distractor condition on the two-item memory condition was significant, *F*(3, 69) = 8.36, *p* < 0.001. Multiple comparisons with the Holm-Sidak method revealed that the RTs in the M1 distractor condition, M2 distractor condition, and New distractor condition were significantly larger than the RTs in the None distractor condition, *t*(23) = 4.87, *p* < 0.001, Cohen’s *d* = 0.18, *t*(23) = 4.47, *p* < 0.001, Cohen’s *d* = 0.21, and *t*(23) = 3.37, *p* = 0.011, Cohen’s *d* = 0.21.

#### FFPs

**Figure 4:**
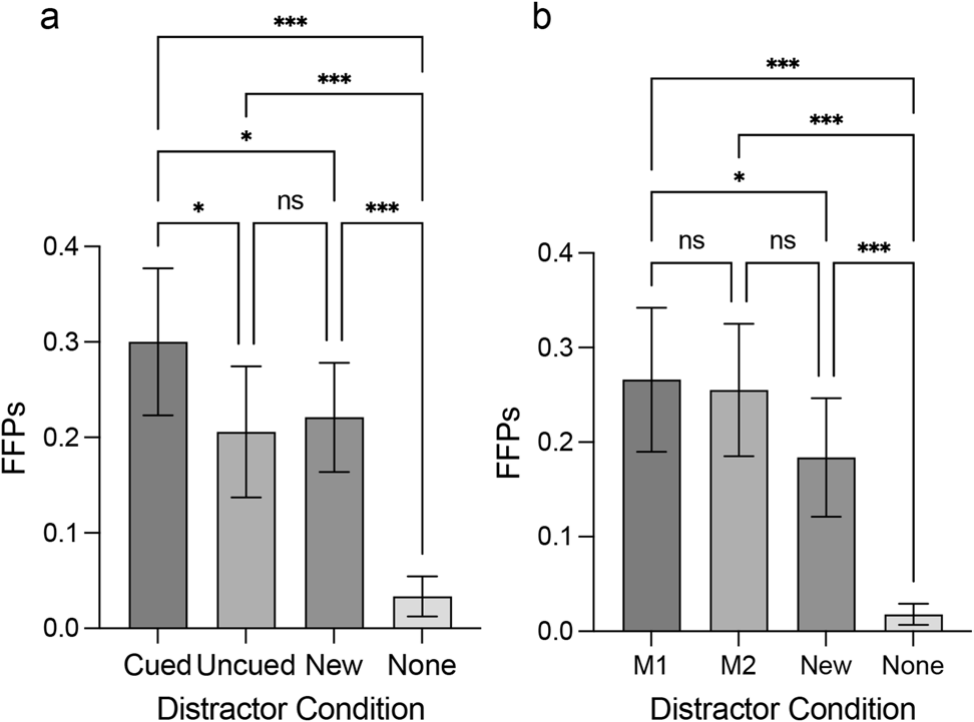
Results of Experiment 2. (**a**) FFPs for the search display as a function of the distractor condition in the one-memory item condition. (**b**) FFPs for the search display as a function of the distractor condition in the two-item memory condition. Error bars indicate 95% CI. *indicates *p* < 0.05, ** < 0.01, *** < 0.001.

Tables 6 and 7 present the descriptive statistics. ANOVA with a Greenhouse–Geisser correction showed a main effect of the distractor condition in the single-item memory condition, *F*(3, 69) = 28.88, *p* < 0.001. Multiple comparisons with the Holm-Sidak method revealed that the FFPs in the Cued distractor condition were significantly larger than the FFPs of the Uncued distractor condition (*t*(23) = 3.20, *p* = 0.012, Cohen’s *d* = 0.09), the New distractor condition (*t*(23) = 3.05, *p* = 0.012, Cohen’s *d* = 0.08), and the None distractor condition (*t*(23) = 7.36, *p* < 0.001, Cohen’s *d* = 0.27). The FFPs from both the Uncued distractor and New distractor condition were significantly larger than the FFPs from the None distractor condition, *t*(23) = 5.15, *p* < 0.001, Cohen’s *d* = 0.17, and *t*(23) = 7.48, *p* < 0.001, Cohen’s *d* = 0.19, respectively (see Fig. 4a).

**Table 6:** FFPs for Experiment 2’s visual search task across all conditions.

Memory condition	Distractor condition	Mean (SD)
Single-item memory	Cued	0.30 (0.18)
	Uncued	0.21 (0.16)
	New	0.22 (0.14)
	None	0.03 (0.05)
Two-item memory	M1	0.27 (0.18)
	M2	0.26 (0.17)
	New	0.18 (0.15)
	None	0.02 (0.03)

**Table 7:** MCI for Experiment 2’s visual search task across all conditions.

Memory condition	Distractor condition	Mean (SD)
Single-item memory	MCI cued	0.26 (0.94)
Two-item memory	MCI M1	0.21 (1.05)
	MCI M2	0.20 (1.01)

There was also a main effect of the distractor condition in the two-item memory condition, *F*(3, 69) = 29.06, *p* < 0.001. Multiple comparisons with the Holm-Sidak method revealed that the FFPs from the M1 distractor condition were significantly larger than the FFPs from the New distractor condition and the None distractor condition, *t*(23) = 3.25, *p* = 0.011, Cohen’s *d* = 0.08, *t*(23) = 6.72, *p* < 0.001, Cohen’s *d* = 0.25. The M2 distractor condition was significantly larger than the FFPs of the None distractor condition, *t*(23) = 2.38, *p* = 0.051, Cohen’s *d* = 0.07, and *t*(23) = 7.05, *p* < 0.001, Cohen’s *d* = 0.24. The New distractor condition was significantly larger than the FFPs from the None distractor condition, *t*(23) = 5.53, *p* < 0.001, Cohen’s *d* = 0.17 (see Fig. 4b).

Experiment 2’s MCI analysis suggests that the combined MCI effect of the M1 and M2 distractors in the two-item memory condition and that of the Cued distractor in the single-item memory condition were not significantly different from each other (*t*(23) = − 0.337, *p* = 0.739, *d* = − 0.15. These results suggest that participants alternated between M1 and M2 as the sole active representation.

As in Experiment 1, we included all data regardless of the exclusion criteria. The results of the FFPs were almost identical to those of the data filtered using the exclusion criteria. See the Supplementary file for further details.

## Discussion

In two pre-registered experiments, the present study investigated whether two VWM representations can be activated alternately to guide attention. The results of the RTs in Experiment 1 suggested that working memory items attracted more attention than non-colour distractors. However, when participants were prevented from predicting the temporal occurrence of the visual search task in the one-item condition of Experiment 2, the results suggested that attentional guidance was not related to working memory. As long as the stimulus had been presented, it would guide attention. In the two-item condition, the results showed that only salient objects within the search display could capture attention. Using eye movements as indicators, we observed that all working memories could capture attention in Experiment 1. The MCI results suggest that the memory items were alternatively activated to influence visual search, such that multiple items shared the same slot available for an active memory item. The results of Experiment 2 were basically consistent with those of Experiment 1. In the two-item condition in Experiment 2, the difference between the M2 distractor and the New distractor was not significant in the two-item condition. Nonetheless, the M2 distractor was essentially the same as the M1 distractor, except one appeared on the left and the other on the right. We believe this resulted from the reduced efficacy of attentional guidance for a single item under the two-item condition. The observation of a reliable and almost consistent result across the two experiments suggests that the memory items were alternatively activated to influence visual search.

We repeated the experiments conducted by Chen and Du^13^. Unfortunately, we could not reproduce their results when RTs were used as an indicator, which could be because they were easily affected by cognitive control^17^. Our experiments required participants to focus their gaze on the center of the screen as much as possible to minimize the gaze already cast on the AOI at the beginning of the search display. This might have changed the participants’ cognitive control, resulting in different results. Further, previous studies found that target-matched visual input enhances the activity of the extrastriate visual cortex while interfering with stimulus-matched visual input suppresses extrastriate visual cortex activity. Thus, suggesting that cognitive control suppresses interference from irrelevant VWM information and promotes tasks concerned with relevant information^23^. The results of the RTs might be the result of inhibiting the input of VWM after early attentional guidance. Therefore, RTs might not effectively reflect the “capturing attention” process. In Experiment 2, participants could not prevent templating in advance, and their cognitive control changed accordingly, leading to the different results of Experiment 2 and Experiment 1.

We also used FFPs, less susceptible to cognitive control than RTs. Consistent with previous eye-tracking studies^19,20^, we observed that two visual working memory representations could capture attention and interfere with concurrent visual search. However, we cannot rule out that memory items are alternatively activated to influence visual search, and we could not conclude that multiple VWM tasks could simultaneously capture attention. To solve this issue, we estimated whether VWM representations could be simultaneously activated to capture attention by calculating the interference caused by distractors and comparing the interference differences between the one-item and two-item conditions. Our results showed that only one working memory item at a time could capture attention, and multiple working memory items could capture attention through alternation. One rational assumption is that the memory item that affects attention is currently being refreshed. According to the time-based resource-sharing theory of working memory, the contents of working memory are continuously refreshed individually to counteract the decline^24^. Selectively boosting the frequency of a memory item and not others creates an imbalance in memory strength that increases the risk of competing with an active item, thereby preventing access to accessory items^25^. Specifically, accessory memory items still need to be refreshed, even though they are weak, and the refreshing frequency is lower than that of the active item. Thus, there is still a chance that attention will be affected. This also explains why both stimuli could be observed to guide attention in Zhang’s research^17^. All experimental stimuli appeared as recognition targets. The stimuli that appeared first in the next task were the active items, and those that appeared later were the accessory items. All the memory items currently refreshed can guide attention, with the active item being refreshed more frequently. Therefore, the probability that an active item is refreshed is greater at the moment of the search display. The probability of guiding attention is also greater than that of accessory items. However, accessory items must be remembered with a low refreshing frequency. Therefore, in the search display, the probability of the accessory item being refreshed is lower than that of the active item. Nevertheless, there is still the possibility of being refreshed and guiding attention. Current research cannot verify this hypothesis, and other possibilities for alternate attentional guidance cannot be ruled out. Whether this alternate attentional guidance is related to refreshing and how exactly it requires further verification. Previous studies proposed that only the representational fidelity of memories, which varies naturally between items, determines whether and how strongly a memory representation guides attention^26,27^. When multiple memory items were maintained, we expected a decrease in fidelity. Our study cannot rule out the possibility that although both working memories can guide attention simultaneously, the guidance effect decreases because of the decrease in any single item in fidelity. This possibility should be explored further in future studies.

The exclusion criteria for the present study deviated from the Stage 1 protocol for several reasons. Our eye tracker (Tobii Pro Nano) had a relatively high error rate when collecting eye-movement data. In addition, as the experiment progressed, participants gradually became inattentive, making it challenging to focus their gaze at the beginning of the search display. Therefore, the range of the AOI set at Stage 1 might not properly reflect the participant’s fixation on the stimulus. The exclusion criteria we designed were too strict, according to which the exclusion rate reached more than 50%. Thus, after consultation regarding this issue, we decided to expand the allowable radius of the central fixation from 3° to 4° and the AOI radius from 1.2° to 1.6°. After this deviation, the exclusion rate was below 40%. While the impact of this deviation on the results is unclear, we made all the data we obtained available, so anyone who cares about this point is free to analyse it.

This study has several limitations. First, there was a relatively high exclusion rate during the experiment. This could be because the experiment was too difficult or the eye tracker lacked sufficient functionality to capture eye movements. However, when we included the full data in the analysis, there was little change in the results, suggesting that the high exclusion rate did not have an overall impact on the study. Second, the verbal coding of the VWM might play a role in affecting attentional guidance. Third, as mentioned earlier, we cannot rule out the possibility that the guidance effect decreases because of a decline in the fidelity of any single item. These limitations should be further explored in future studies.

Notwithstanding, our results suggest that only one working memory item at a time affects attention, and multiple working memory items may affect attention through alternation. Further research is needed to determine how to carry out alternations.

### Supplementary Information

Supplementary Information.

## Data Availability

Raw data are available at the OSF (https://osf.io/twcvs). See the manual for more details (https://osf.io/pjdt9).

## Code availability

All codes and materials are available at OSF (https://osf.io/qmr67). See the manual for more details (https://osf.io/pjdt9).
